# Breed Distribution of the Superoxide Dismutase 1 Gene Polymorphism Associated With Degenerative Myelopathy in a Canine Population From Different Geographical Regions of Türkiye

**DOI:** 10.1002/vms3.70725

**Published:** 2026-03-14

**Authors:** Merve Yüksel, Kerem Ege, Ilayda Karakuş, Bilal Akyüz

**Affiliations:** ^1^ Department of Veterinary Genetics and Biotechnology Institute of Health Science Erciyes University Kayseri Türkiye; ^2^ Department of Veterinary Genetics Fırat University Elazığ Türkiye; ^3^ Faculty of Veterinary Medicine Fırat University Elazığ Türkiye; ^4^ Department of Veterinary Genetics Erciyes University Kayseri Türkiye

**Keywords:** degenerative myelopathy, German Shepherd, Kangal Shepherd, restriction fragment length polymorphism‐polymerase chain reaction, SOD1 gene, Toy Poodle

## Abstract

**Background and Objective:**

Canine degenerative myelopathy (CDM) is a progressive neurodegenerative condition that impacts the spinal cord, resulting in paralysis in dogs. CDM is linked to a missense mutation in the superoxide dismutase 1 (*SOD1*) gene. This study aimed to examine the presence of the SOD1:c.118G>A mutation in the German Shepherd, Golden Retriever, Pomeranian, Toy Poodle and native Kangal Shepherd dog breeds in Türkiye.

**Methods:**

Blood samples (*n* = 161) from five breeds, collected from six provinces across five geographic regions of Türkiye, were tested for the prevalence of the mutant allele associated with CDM. All dogs were clinically healthy and sampled for genetic testing. Genotyping was performed using the PCR‐RFLP method and gel electrophoresis.

**Results:**

Genotyping of dogs revealed that 141 had the homozygous wild‐type genotype (GG), 20 were heterozygous carriers (AG), and there were no homozygous mutant (AA) individuals. The mutant A allele was determined in Kangal Shepherd, German Shepherd and Toy Poodle dog breeds. The frequency of the mutant allele in the investigated population was identified as 0.062. It was determined that the mutant allele, which was not previously reported in the Kangal Shepherds, was present, and the mutant allele frequency revealed 0.057.

**Conclusions:**

The SOD1:c.118G>A mutation is present in dog breeds in Türkiye, and is also detected in the Kangal Shepherd, an ancient breed. This finding underscores the importance of genetic testing in dogs, as it is crucial to prevent the spread of mutations in different canine populations. Carrier dogs can be clinically identified to prevent breeding and reduce CDM incidence.

## Graphical abstract

The SOD1:c.118G>A mutation associated with canine degenerative myelopathy was identified for the first time in the Kangal Shepherd, with an allele frequency of 0.057; it was also detected in the German Shepherd at a frequency of 0.140 and in the Toy Poodle at 0.089. The mutant allele frequency in the canine population in Türkiye has been estimated at 0.062.

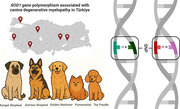

## 1 Introduction

The domestic dog (*Canis lupus familiaris*), one of the first domesticated species, has more than 400 breeds today, developed through human breeding and selection practices (Galibert et al. 2011). The development of strict breed standards using inbreeding tactics in developing these breeds made it easier to identify preferred alleles. Still, this practice increased the prevalence of autosomal recessive genetic disorders (Zierath et al. 2017).

Canine degenerative myelopathy (CDM), a fatal progressive neurodegenerative disorder leading to adult‐onset motor neuron loss and paraplegia in affected dogs, was first reported in German Shepherd dogs in 1973 (Averill 1973). It was later reported that this hereditary disorder was also found in various breeds such as the Siberian Husky, Pembroke Welsh Corgi and Boxer (Bichsel et al. 1983; Coates et al. 2007; Miller et al. 2009). Clinical signs in dogs with CDM usually begin at 8 years of age or older, and the disorder is sex‐independent (Coates and Wininger 2010). The prognosis of the disorder is relatively poor. Within 1 year of the onset of clinical signs, mainly when paralysis of the lower body occurs, dog owners often opt for euthanasia (Coates and Wininger 2010). Sometimes, rapidly progressive respiratory dysfunction causes death in affected animals (Oyake et al. 2016). There is currently no established treatment protocol for CDM, so preventing carrier × carrier mating seems to be the best option to combat this inherited disorder (Neeves and Granger 2015).

In the Online Mendelian Inheritance in Animals database (OMIA 2025), approximately 450 single‐gene disorders were identified in different dog breeds. Furthermore, dogs are an essential animal model for human genetic disease because of causal variants in human genes (Shaffer et al. 2019). Degenerative myelopathy, which is similar to human amyotrophic lateral sclerosis (ALS) in terms of clinical presentation and genetics, shows an autosomal recessive mode of inheritance (Nardone et al. 2016), occurs as a result of a mutation causing a G‐to‐A substitution (c.118G>A) in Exon 2 of the superoxide dismutase (*SOD1*) gene (OMIA mutation ID 36; OMIA phene ID 000263–9615) (OMIA 2025). This gene is located on Chromosome 31 (CFA31) and encodes the SOD1 protein, one of the most abundant proteins in the central nervous system, and plays a crucial role in free radical removal (Awano et al. 2009). The mutated *SOD1* gene, which is associated with CDM, is also associated with ALS cases in humans (Golubczyk et al. 2019). Mutant *SOD1* leads to a change from glutamate to lysine (p.E40K) at the 40th amino acid of the SOD polypeptide chain (Awano et al. 2009). Toxicity due to conformational changes in the E40K mutant protein plays a role in the aetiology of degenerative myelopathy (Gouveia et al. 2023).

In 2018, the Fédération Cynologique Internationale (FCI) recognised the Kangal Shepherd Dog as a native Turkish breed (Standard No: 331). It is well established that the Turkish communities migrating from Central Asia, whose primary livelihood depended on nomadic animal husbandry, brought their strong livestock guardian dogs (FCI 2025). Based on phylogenetic analyses, the closest breeds to the Kangal Shepherd breed are the Scandinavian and Southwest Asian (Afghanistan, Uzbekistan, Tajikistan and Kazakhstan) (Koban et al. 2009). The Kangal Shepherd breed has maintained its original characteristics without deterioration, primarily due to its widespread utilisation by sheep breeders and its exceptional vigilance against wolf predation (Kinka and Young 2019; Akyazi et al. 2018). The Kangal Shepherd is considered the most well‐known dog in Türkiye and is mainly preferred for herd protection and guard duties (Dimitrijevic et al. 2020). Although popular in Türkiye, Kangal Shepherd dogs are also bred in most European countries, including Belgium, France, Germany and Slovenia (Yılmaz and Ertuğrul 2015). This prevalence may be attributed to the utilisation of the Kangal Shepherd in maintaining the lineage of various breeds due to its high adaptability and resilience to diverse climatic conditions (Koçkaya et al. 2019). As in other dog breeds, measures must be implemented to identify and prevent genetically transmitted disorders while preserving the desired phenotypic characteristics in the Kangal Shepherd.

While various studies of CDM in different dog breeds have been performed (Majchrakova et al. 2023; Kountourantzis et al. 2023; Ito et al. 2024; Moretti et al. 2024), data on dog breeds in Türkiye are relatively scarce. The related variants could be valuable markers for breeding strategies intended to reduce the incidence of CDM. Significant breeds such as the German Shepherd, Golden Retriever, Pomeranian and Toy Poodle, although not indigenous to Türkiye, are among the most commonly owned companion dog breeds in the country. Investigation of the CDM‐associated mutation in these breeds bred in Türkiye is expected to increase awareness among breeders and veterinarians, while generating essential epidemiological data on breed‐specific mutation frequencies to inform disease management strategies. Therefore, this study aimed to determine the frequency of the c.118G>A mutation in German Shepherd, Golden Retriever, Pomeranian, Toy Poodle and native Kangal Shepherds dog breeds from different regions of Türkiye.

## 2 Materials and Methods

### 2.1 Study Period and Location

**FIGURE 1:**
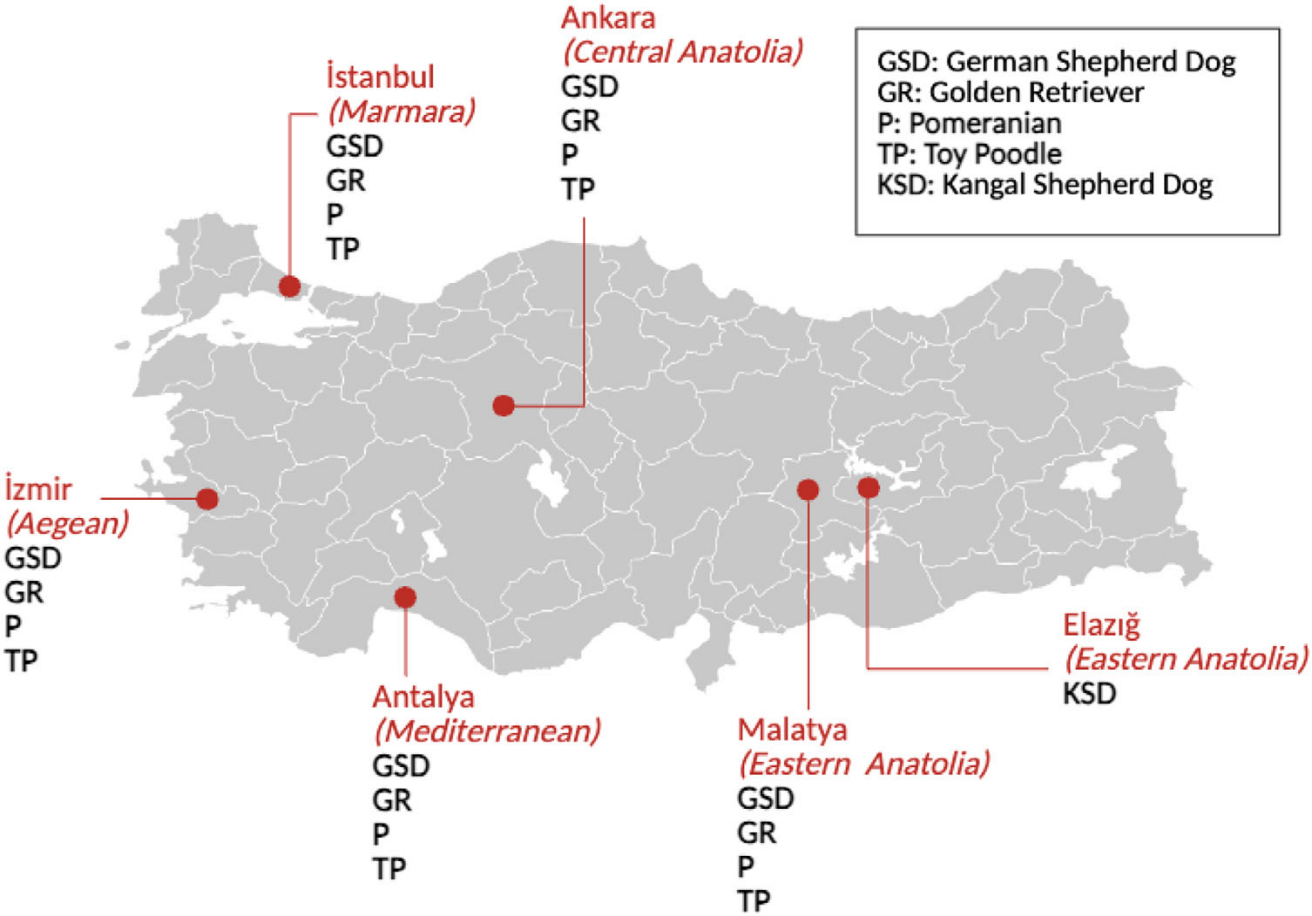
A map of the study population (*N* = 161) consisted of five dog breeds in various provinces, representing different geographical regions of Türkiye.

Between June 2024 and March 2025, blood samples were collected from 25 German Shepherds, 20 Golden Retrievers, 18 Pomeranians, 28 Toy Poodles and 70 native Kangal Shepherds. The dogs were randomly included, considering only their breed. In this study, the research population consisted of dogs in six provinces representing five geographical regions of Türkiye (Marmara, Aegean, Mediterranean, Central Anatolia and Eastern Anatolia Regions) (Figure 1).

### 2.2 Sample Collection

Breed selection was based on the popularity of certain dog breeds in Türkiye, as well as breeds that we considered would provide valuable epidemiological insights regarding CDM. The total number of dogs included per breed was determined based on breed representation and their availability from owners, a breeder and clinics, with none of the animals having any suspected CDM diagnosis. The German Shepherds, Golden Retrievers, Pomeranians and Toy Poodles included in the study were healthy dogs presented to veterinary clinics across different provinces of Türkiye for routine vaccinations and health check‐ups. Additionally, samples from native Kangal Shepherds were collected from a purebred breeder located in Elazığ province and the animals were reported as healthy by the responsible veterinarian. The Kangal Shepherd dog breeder maintained the continuity of their herds by introducing breeding stock from different kennels within the country. There were no exclusion criteria based on the sex or age of the dogs, and the research population consisted of 95 female and 66 male dogs, ranging in age from 2 months to 15 years (Table 1). Blood samples (2–4 mL) were taken using a sterile double‐ended cannula from the left or right cephalic vein of each dog into vacuum‐sterile EDTA blood tubes (Vacuette, Greiner Bio‐One, Kremsmünster, Austria). The collected blood samples were transported in insulated boxes with ice packs at 4°C until they reached the analysis laboratory within 1–2 days. The blood samples were kept in the refrigerator at −20°C for DNA isolation.

**TABLE 1:** Distribution of dogs by breed, age and sex included in the study for screening the SOD1:C.118G>A mutation associated with CDM.

Breed	Age	Number of dogs
Kangal Shepherd	6 months–9 years	70 (Female: 37, male: 33)
German Shepherd	2 months–6 years	25 (Female: 16, male: 9)
Golden Retriever	2 months–15 years	20 (Female: 14, male: 6)
Pomeranian	2 months–10 years	18 (Female: 12, male: 6)
Toy Poodle	1–12 years	28 (Female: 16, male: 12)
Overall		161 (Female: 95, male: 66)

### 2.3 DNA Isolation

Genomic DNA was extracted from peripheral whole blood using the standard phenol/chloroform‐based method. After isolation, DNA samples were stored at −20°C. For assessment of the quality of these isolated DNA samples, agarose (AgaPure Agarose LE) gel electrophoresis (Bio Rad Laboratories, California, USA) was carried out, and samples were then visualised using a UV transilluminator (ECX‐20‐M, Vilber Lourmat, Marne‐la‐Vallée, France). The concentrations of isolated DNA samples were determined by measuring optical density at 260/280 nm through a NanoDrop 2000/2000c Spectrophotometer (Thermo Fisher Scientific, Waltham, Massachusetts, USA).

### 2.4 PCR‐Restriction Fragment Length Polymorphism (RFLP) Analysis

PCR was performed to investigate the presence of the mutation causing CDM in the canine *SOD1* gene using primers CDM_F (5′‐AGTGGGCCTGTTGTGGTATC‐3′) and CDM_R (5′‐TCTTCCCTTTCCTTTCCACA‐3′) developed by Holder et al. (2014). A 292 bp section in Exon 2 of the canine *SOD1* gene was amplified by the PCR. The PCR mixture was carried out in a total volume of 20 µL, containing 1.5 mm MgCl_2_, 250 µm dNTPs, 200 µm of each primer, 10 × PCR buffer (Thermo Fisher Scientific, Waltham, Massachusetts, USA), 1.5 U Taq polymerase (Thermo Fisher Scientific, Waltham, Massachusetts, USA), 50 ng DNA template and deionised water. The PCR conditions for CDM mutation detection included pre‐denaturation at 95°C/10 min, followed by 37 cycles consisting of denaturation at 94°C/40 s, annealing at 55°C/30 s, 72°C/1 min, and post‐elongation at 72°C/10 min. PCR products were detected by electrophoresis in 2% agarose (AgaPure Agarose LE) gels stained with ethidium bromide (EtBr). The PCR products were digested using 5 U of the *Acu*I restriction enzyme (Thermo Fisher Scientific, Waltham, Massachusetts, USA) at 37°C for at least 55 h, followed by the inactivation of the enzyme at 65°C for 20 min. RFLP products were detected by electrophoresis in 3% agarose (AgaPure Agarose LE) gels stained with ethidium bromide (EtBr). After digestion of PCR products with *Acu*I restriction enzyme, two bands (230 and 62 bp) were expected in dogs with normal genotype (GG), three bands (292, 230 and 62 bp) in heterozygous dogs (AG) and only one 292 bp band in dogs homozygous for the CDM‐associated mutation (AA). PCR and RFLP products were visualised with a UV transilluminator (ECX‐20‐M, Vilber Lourmat, Marne‐la‐Vallée, France).

### 2.5 Statistical Analysis

Total sample size was determined based on a power analysis for the chi‐square (χ^2^) test using G*Power software (version 3.1), assuming an effect size of 0.3, Type I error probability (*α*) of 0.05 and a power of 95% [Power (1–*β*)]. The allele and genotype frequencies of the population were calculated using Microsoft Excel. The Hardy–Weinberg equilibrium was calculated using a chi‐square (χ^2^) test by comparing observed and expected genotype frequencies for each breed, with the aid of an online tool (http://apps.biocompute.org.uk/).

## 3 Results

**FIGURE 2:**
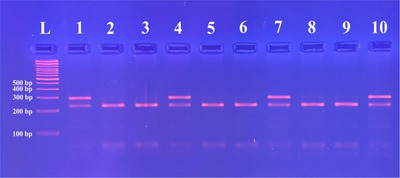
Agarose gel electrophoresis of the PCR‐RFLP products of representative samples. Lane L: 100 bp DNA Ladder (Thermo Fisher Scientific, Waltham, Massachusetts, USA). Lanes 2, 3, 5, 6, 8 and 9: GG genotype (230 and 62 bp). Lanes 1, 4, 7 and 10: AG genotype (292, 230 and 62 bp).

The obtained 292 bp PCR products were digested by *Acu*I restriction endonuclease. As a result of the genotyping of the breeds, it was observed that 141 out of 161 dogs examined did not carry the mutant allele (A) and had a homozygous wild‐type genotype (GG); 20 were heterozygous (AG) for the mutant allele. No homozygous (AA) individuals for the mutant allele were encountered in the examined samples. Genotype patterns of the examined samples from five dog breeds in terms of CDM are shown in Figure 2.

### 3.1 Genetic Analysis

The mutant A allele was identified in three of the five breeds, with the genotype distribution across all breeds shown in Table 2.

**TABLE 2:** Genotype and allele frequencies of the SOD1:C.118G>A mutation associated with CDM in five dog breeds.

Breed	Genotype frequencies			Allele frequency		χ2 *p* value
	GG	AG	AA	A	G	
Kangal Shepherd	0.886 (*n* = 62)	0.114 (*n* = 8)	0 (*n* = 0)	0.057	0.943	0.612 ns
German Shepherd	0.720 (*n* = 18)	0.280 (*n* = 7)	0 (*n* = 0)	0.140	0.860	0.416 ns
Golden Retriever	1 (*n* = 20)	0 (*n* = 0)	0 (*n* = 0)	0	1	—
Pomeranian	1 (*n* = 18)	0 (*n* = 0)	0 (*n* = 0)	0	1	—
Toy Poodle	0.821 (*n* = 23)	0.179 (*n* = 5)	0 (*n* = 0)	0.089	0.911	0.604 ns
Overall	0.876 (*n* = 141)	0.124 (*n* = 20)	0 (*n* = 0)	0.062	0.938	0.401 ns

*Note*: χ2, chi‐squared test; ns, not significant; *n*, number of dogs; *p*, probability value.

The observed frequencies of the three genotypes, calculated under the assumption of Hardy–Weinberg equilibrium (HWE), are shown in Table 2. No significant departure from HWE was detected. Breed‐specific differences were noted in the detection of the mutant A allele (Table 2). The mutant A allele was detected in Kangal Shepherds, German Shepherds and Toy Poodles at frequencies of 0.057, 0.140 and 0.089, respectively.

## 4 Discussion

Diagnosis of CDM in dogs can involve various approaches, including spinal magnetic resonance imaging and cerebrospinal fluid analysis; however, genetic testing of the SOD1 mutation is both practical and reliable, and is relatively more commonly used for screening and confirming the disease (Bouché et al. 2023). This study screened five of the most popular dog breeds in Türkiye, and to our knowledge, it represents the first report of CDM screening in these breeds within the country. This was also the first CDM screening of purebred native Kangal Shepherd in Türkiye. Additionally, the nearly homogeneous distribution of the examined dog population across five distinct geographical regions of Türkiye may provide a reasonable representation for CDM screening efforts within the country.

As of now, the mutation linked to CDM in Kangal Shepherd dogs has been explored solely by Cocostîrc et al. (2023), who stated that the one dog analysed in the study did not possess the pertinent mutation. In the Kangal Shepherd dogs analysed in this study, the mutant allele frequency was determined to be 0.057, representing a first report of the mutant allele in this breed. Additionally, the observed mutant allele frequency can be considered an initial step toward understanding the CDM‐associated genetic structure in the Kangal Shepherd breed in Türkiye.

CDM is extensively documented in German Shepherd dogs, as it was the first breed in which the condition was identified. In the current study, the frequency of the mutant allele in the German Shepherd breed was calculated as 0.14. The studies conducted in Mexico and Greece also reported similar frequencies for the German Shepherd breed, which were 0.13 and 0.14, respectively (Ayala‐Valdovinos et al. 2018; Kountourantzis et al. 2023). In another study, investigating the German Shepherd population in Belgium, the Netherlands and Germany, the allele frequency was calculated as 0.15 (Broeckx et al. 2013). Mutant allele frequencies in the German Shepherd breed have been reported as 0.20 in Romania (Cocostîrc et al. 2023), 0.22 in Japan (Maki et al. 2022), 0.29 in Italy (Ghilardi et al. 2024), 0.37 in the USA (Zeng et al. 2014), 0.12 in Brazil (Santos et al. 2020), 0.15 in Paraguay and 0.23 in Uruguay (Artigas et al. 2024). While a relatively high mutant allele frequency of 0.35 was reported in German Shepherd dogs in the United Kingdom (Holder et al. 2014), a more recent study indicated a lower frequency of 0.20 (Donner et al. 2023). Relatively high allele frequencies have been reported in German Shepherd dogs, which could pose challenges for strict restrictions on breeding carrier individuals (Holder et al. 2014). The mutant allele frequency observed in German Shepherds in the present study is comparable to that reported in other populations (Broeckx et al. 2013; Ayala‐Valdovinos et al. 2018; Kountourantzis et al. 2023), suggesting that the prevalence of the CDM‐associated mutation may be broadly consistent across different geographical regions.

For Toy Poodles, the mutant allele frequency determined by Zeng et al. (2014) was 0.13 based on the analysis of 4 dogs, whereas in this study, it was 0.089 based on 28 dogs. In a recent study, Ghilardi et al. (2024) reported a mutant allele frequency of 0.06 in Poodles. The higher mutant allele frequency reported by Zeng et al. (2014) may have been influenced by the relatively small population of 4 dogs analysed, while the larger sample of 28 dogs in the present study may have provided a slightly lower and potentially more representative estimate in Toy Poodles.

For Golden Retrievers and Pomeranians, the mutant allele frequencies determined by Zeng et al. (2014) were 0.03 and 0.12, respectively, whereas in the current study, no mutant allele was detected in these breeds. A similar conclusion was reached by Ito et al. (2024) and Broeckx et al. (2013), both of whom reported no presence of the mutant allele in Golden Retrievers, consistent with the findings of the present study. These findings indicate that the mutant allele is infrequently observed in Golden Retrievers. However, data from additional populations are needed to confirm these observations.

In a screening of 22 dog breeds in Mexico, the mutant A allele was identified in 11 different dog breeds (Ayala‐Valdovinos et al. 2018). In a screening of 8 of 26 dog breeds in Romania, the mutation was identified for the first time in the Romanian Mioritic Shepherd in addition to the Wire Fox Terrier, German Shepherd, Rottweiler, Belgian Shepherd and Czechoslovakian Wolfdog (Cocostîrc et al. 2023). In 2014, a comprehensive analysis genotyped 33,746 dogs from 56 breeds for the SOD1:c.118G>A mutation linked to CDM, revealing that the mutant frequency ranged from 0.94 to 0.01 among the studied breeds (Zeng et al. 2014). The highest frequencies were observed in the Wire Fox Terrier, Pembroke Welsh Corgi, Boxer and Cavalier King Charles Spaniel compared to other breeds (Zeng et al. 2014). The study, based on owner surveys of 512 dogs, estimated that 60% of dogs homozygous for the mutant allele exhibited clinical signs of CDM. In contrast, heterozygous dogs had a risk similar to that of those with the homozygous genotype for the normal allele.

This study and other researchers, such as Zeng et al. (2014) and Kountourantzis et al. (2023), show that the SOD1:c.118G>A mutation is not breed‐specific. Additionally, in agreement with the findings of Zeng et al. (2014), our results indicate that the mutant allele associated with CDM can be found not only in medium and large breeds but also in small breeds, such as the Toy Poodle, contrary to the common assumption. Additionally, in agreement with the findings of Zeng et al. (2014), our results indicate that the mutant allele associated with CDM can also occur in small breeds, such as the Toy Poodle, suggesting its potential presence beyond medium and large breeds and highlighting the need for studies in larger populations to better understand its distribution across different breeds.

It is crucial to prevent mating between carriers to avoid producing homozygous affected offspring for the autosomal recessive mutation associated with CDM. Consequently, parents should undergo DNA‐based genetic testing, as demonstrated in this study. Moreover, if a dog carrying the mutant allele possesses desirable traits, breeding with another carrier can be prevented (O'Brien and Leeb 2014). To ensure the preservation of desirable traits and other essential genetic factors, decisions based on this information must be made rationally and ethically (O'Brien and Leeb 2014).

It is crucial to clinically distinguish CDM from other conditions with similar neurological pathologies, such as Intervertebral Disc Disease (IVDD), as misdiagnosis may lead to inappropriate surgical interventions (Coates and Wininger 2010; Santos et al. 2020). Genetic testing for the SOD1:c.118G>A mutation can distinguish diagnostic from other possibilities for CDM; moreover, it can provide early insight into the risk of developing the disorder. A study conducted in the United Kingdom reported that while some dogs aged over 8 years with the AA genotype for the SOD1:c.118G>A mutation have pelvic limb ataxia, the AA genotype was absent in dogs with non‐neurological conditions (Holder et al. 2014). Nevertheless, veterinarians should interpret such genetic tests cautiously, as a dog carrying mutations in both alleles and having the CDM phenotype may still be affected by a different disease (Kountourantzis et al. 2023). The absence of homozygous AA genotypes in the samples from the breeds analysed in this study is thought to be partly due to the investigation of ostensibly healthy dogs and the relatively limited sample sizes within each breed.

In this study, the report of the mutant allele associated with CDM in the Kangal Shepherd, German Shepherd and Toy Poodle populations shows the need for studies related to this genetic disorder in Türkiye. Zeng et al. (2014) conducted a comprehensive study on the prevalence of this hereditary condition across 222 dog breeds and also cross‐bred dogs, excluding the Kangal Shepherd. The study found the mutant A allele in 124 breeds as well as in cross‐bred dogs. As this hereditary disorder is not breed‐specific, preventing mating between carriers across all breeds is essential to reduce the risk of affected individuals. Moreover, because the disorder's symptoms appear later in life, after affected animals reach sexual maturity, CDM‐affected dogs can transmit the mutant allele to their offspring when mated. This highlights the importance of detecting carriers and affected animals before sexual maturity to prevent breeding.

Epidemiological data on genetic disorders in different breeds are crucial for developing prevention strategies and sharing these findings globally is essential for understanding the allele distribution of specific disorders (Santos et al. 2020). On the other hand, its occurrence not only in today's dog breeds but also in Kangal Shepherd breed, the majority of which have been developed in the last 100–150 years and whose origins are said to date back to the ancient Hittite civilisation (Daşkıran and Ertuğrul 2004), raises doubts about the transfer of the SOD1:c.118G>A mutation from its primitive ancestors to domestic dogs. To clarify this issue, we think it is necessary to investigate the presence of the SOD1:c.118G>A mutation in ancient dog breeds in Eurasia. While this study provides valuable insights, future research, including a larger number of dogs per breed and additional breeds, could offer an even more comprehensive understanding. Additionally, although the examined Kangal Shepherd population included breeding animals from different kennels, the presence of half‐siblings among some individuals represents a potential limitation; nevertheless, our study provides preliminary evidence that the CDM‐associated variant may trace back to ancient breeds, offering a basis for future, more detailed investigations.

## 5 Conclusion

This study is the first to report the presence of the mutant allele responsible for CDM in the Kangal Shepherd dog. Consequently, it indicates that further research on the SOD1:c.118G>A mutation is needed across all dog breeds, including ancient ones. Both the current and previous studies have shown that parents should be screened for the SOD1:c.118G>A mutation to prevent carrier–carrier mating in breeds where this mutation has been reported.

This study highlights the necessity of additional investigation on the SOD1:c.118G>A mutation in ancient dog breeds and is expected to improve understanding of the genetic lineage of the Kangal Shepherd.

## Author Contributions

**Merve Yüksel**: Writing – review & editing; resources, formal analysis, visualization, methodology, conceptualization. **Kerem Ege**: Sample collection, methodology, funding acquisition. **İlayda Karakuş**: Sample collection, methodology, acquisition of financing. **Bilal Akyüz**: Writing – review & editing, supervision, conceptualization, validation, funding acquisition.

## Funding

This research was supported by the 2209‐A Tubitak (Scientific and Technological Research Council of Türkiye) Project (Grant number: 1919B012335774).

## Conflicts of Interest

The authors declare no conflicts of interest.

## Ethics Statement

The study protocol was approved by the Fırat University Animal Experiments Local Ethics Committee (Approval No: 2025/09‐08). All dogs have informed breeder/owner consent. All samples were non‐invasive whole blood samples on EDTA tubes.

## Data Availability

The data are available upon request from the authors.
